# Use of LUCS (Light-Up Cell System) as an alternative live cell method to predict human acute oral toxicity

**DOI:** 10.1016/j.toxrep.2020.02.010

**Published:** 2020-02-19

**Authors:** C. Gironde, C. Dufour, C. Furger

**Affiliations:** Anti Oxidant Power, AOP/MH2F team, LAAS/CNRS, 7 avenue du Colonel Roche, BP 54200, Toulouse Cedex, 31031, France

**Keywords:** Toxicity testing, Alternative methods, Non-animal testing, Basal cytotoxicity

## Abstract

LUCS (Light-Up Cell System) is a new live cell test that allows assessment of a cell’s homeostasis and its alteration by a toxic agent. To evaluate the effectiveness of LUCS as an alternative test method for acute oral toxicity, we compared EC_50_s determined in HepG2 cells treated with 53 chemicals selected from the ACuteTox EU database with corresponding human blood LC_50_s derived from human acute poisoning cases. Linear regression analysis showed that LUCS results predict human data to 69 %. Rodent oral LD_50_s and LUCS EC_50_s were then correlated to human LC_50_s using shared data sets. Linear regression analyses comparing LUCS and animal data clearly showed that LUCS always predicts human toxicity better than animal data do.

These successful prediction values prompted us to simplify the LUCS test, adapting it to regulatory and high throughput applications, resulting in a new protocol with consistent dose-response profiles and EC_50_s.

This study demonstrates that the LUCS test method could be relevant for assessing human acute oral toxicity with a simplified protocol adapted to commercially available fluorescence readers. We suggest that this new alternative method can be used for acute systemic toxicity testing in combination with other tests under European REACH and other regulations, wherever pertinent alternative methods are still lacking.

## Highlights

• Innovative LUCS assay, based on human cells, showed relevant human acute oral toxicity prediction values.
• LUCS predicts human data to 69 %, better than animal data do.
• A new protocol is proposed, allowing an easy implantation on fluorescence readers for high throughput applications.
• LUCS test method is fast and robust, and can be used in multiplex analyses.

## Graphical abstract

**Figure:**
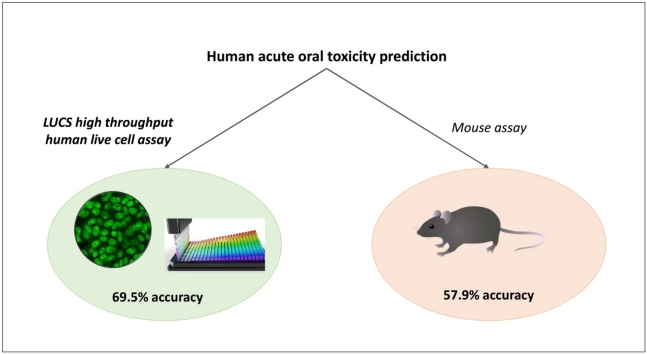

## 1 Introduction

Intensifying demands to address the 3Rs concept (Reduction, Refinement, and Replacement of the use of animals in research) introduced by Russel and Burch [1] has increased the push for new screening strategies into public and private scientific research. However, despite the 3Rs incorporation into the European legislation (directive 2010 /63) [2], that replaces and repeals 1986’s directive 86/609/EEC), and the emergence of a new toxicology paradigm (*i.e.* more relevant to humans and based on molecular and cellular pathways [[3], [4], [5], [6], [7], [8]]), the progress of alternative methods into regulatory use has been slow [9,10]. Except for the cosmetic products regulation where animal tests have been banned since 2013 (regulation No 1223/2009 [11]), safety and regulatory testing still rely on animal tests. However, a U.S. national strategy and roadmap for implementing non-animal approaches to assess potential hazards associated with acute exposures was recently launched by ICCVAM and other U.S. agencies [12,13].

To date, acute systemic toxicity data, in particular those required for hazard classification and substance labelling, are informed by animal tests. In Europe, the regulatory requirements for acute toxicity testing of substances are outlined in several regulations: 1) REACH (Registration, Evaluation, Authorisation and Restriction of Chemicals) Regulation No 1907/2006 to improve human health and environment protections [14]; 2) the CLPR (Classification and Labelling and Packaging Regulation) No 1272/2008 to ensure that chemical hazard is clearly communicated to workers [15]; 3) the PPPR (Plant Protection Products Regulation) No 1107/2009 which applies to insecticides, fungicides, herbicides [16]; 4) the BPR (Biocidal Products Regulation) No 528/2012 which applies to bio-active substances [17]. Currently, acute systemic toxicity is assessed in rodents by oral, dermal or inhalation exposure in accordance with the OECD test guidelines (TGs). For acute oral toxicity tests, TG 420, TG 423, and TG 425 [[18], [19], [20]], each uses 5–9 animals. For acute inhalation, TG 403 uses at least 20 animals, TG 436, 6–12 animals, and TG 433, 5–10 animals [[21], [22], [23]]. For acute dermal route, TG 402 uses about 10 animals [24]. Results expressed as the median Lethal Doses (LD_50_, doses in mg/kg of animal body weight that kill 50 % of the tested animals) are used to assign substances to different toxicity categories, determining the hazard labelling on manufactured products that inform human users. Apart from the obvious ethical aspects, the limitations of animal models and the validity of extrapolating animal data to human effects have been widely discussed, especially in terms of reproducibility and relevance issues [25,26].

Alternative *in vitro* methods have been validated and adopted as TG by the ODCE (reviews in [[27], [28], [29]]). Efforts have however essentially focused on the less demanding domains of acute topical toxicities or local tolerance *i.e.* phototoxicity (TG 432, TG 495), skin corrosion/irritation (TGs 430, 431, 435/439), serious eye damage/irritation (TGs 437, 492, 438, 491, 460), and skin sensitisation (TGs 442C, 442D, 442E, see GD263) (https://www.oecd-ilibrary.org/environment/oecd-guidelines-for-the-testing-of-chemicals-section-4-health-effects_20745788), which represent only a small portion of the test needs. Also, for systemic toxicity testing (which includes acute toxicity, carcinogenicity, genotoxicity, reproductive toxicity, and repeated dose toxicity), alternative *in vitro* methods have been adopted by the OECD. They are dedicated in genotoxicity testing (TGs 471, 476, 490, 473, 487) or mechanistic screening of substances with a potential endocrine disruptor function (TGs 455, 456, 457, 458, 493), and thus only partially replace animal *in vivo* tests.

In the domain of acute systemic toxicity, the use of cell-based test methods to predict acute oral systemic toxicity in humans have been extensively investigated by the MEIC [[30], [31], [32]] and ACuteTox [33] programmes. These programmes have shown a correlation between *in vitro* and *in vivo* toxicity. Two main studies that evaluate the usefulness and limitations of an *in vitro* cytotoxicity test method have led to the validation of a cytotoxicity test for potential adoption by regulatory authorities. Firstly, a multi-laboratory validation study performed by ICCVAM/NICEATM and ECVAM laboratories used neutral red uptake (NRU) readout in BALB/c 3T3 mouse fibroblasts (3T3) and normal human epidermal keratinocytes (NHK) on 72 reference substances. The resulting data [34,35] showed a correlation between EC_50_ and oral LD_50_ data, with poor prediction of estimated individual LD_50_ values, however. It was concluded that NHK- and 3T3-NRU could be applied, if only to estimate the starting doses used for performing the rodent acute oral systemic toxicity tests [36]. Secondly, as predictive capacity was better for low rather than high toxicity compounds, a follow-up validation study based on 56 substances was coordinated by the EURL ECVAM and two other laboratories (HSL, UK and IIVS, USA) to estimate the predictive capacity of the 3T3 NRU test in order to discriminate between classified (LD_50_ < 2000 mg/kg b.w.) and unclassified substances (LD_50_ > 2000 mg/kg b.w.) [37]. Conclusions were published by the EURL-ECVAM as recommendations on the use of 3T3 NRU test method [38].

To summarise, 3T3 NRU test can be used but only to identify substances not requiring classification for acute oral toxicity (above 2000 mg/kg b.w. threshold). For regulatory use and in the context of REACH, this alternative method may thus be used in combination with other information sources when building a Weight-of-Evidence (WoE) case, or as a component of an Integrated Approaches to Testing and Assessment (IATA), but only to substances of low acute oral toxicity (*i.e.* not classified) (see ECHA chapter R.7.4. 3.1.2. in https://echa.europa.eu/documents/10162/13632/information_requirements_r7a_en.pdf/e4a2a18f-a2bd-4a04-ac6d-0ea425b2567f).

Toxicity pathways that may lead to acute systemic toxicity are numerous and an increasing number of new AOPs (Adverse Outcome Pathways) is being reported (https://aopwiki.org/). Toxicity is described either as resulting from general cytotoxic mechanisms caused by interference with cellular basic structures or functionally important components (cell membrane permeability, metabolism, ion regulation, cell division, oxidative stress) or resulting from specific cell/organ/system toxicity mechanisms (affecting a specific ion channel, CNS- or a cardiac-receptor). Nevertheless, it appeared that most chemicals with specific effects on target organs were classified by the *in vitro* 3T3 NRU assay as acutely toxic by basal cytotoxicity [39], confirming general toxicity as an important determining factor of acute systemic toxicity.

Here, we present a new cell-based approach called LUCS and report that regression analysis of human acute lethal blood concentrations with *in vitro* LUCS data resulted in a better correlation than with standard *in vivo* rodent oral lethal doses. In other words, LUCS data predict human toxicity better than animal data do. Aware of limitations in LUCS procedure, we also present an optimization of the protocol allowing the LUCS test to be easily implemented on any commercially available fluorescence reader. This study demonstrates both suitability and benefit of the LUCS method test as a dedicated test of acute oral toxicity for both regulatory purposes and industrial high throughput applications.

## 2 Materials and methods

### 2.1 Selection of chemicals

53 chemicals were selected from the ACuteTox database (97 reference chemicals, www.acutetox.eu) on the criterium of commercial availability. The compounds described in Table 1.S. (Supplementary data) included pharmaceutical drugs (30 substances), industrial chemicals (12 substances), and biocides (11 substances). The 53 compounds have been assigned to their different toxicity categories according to the EU Classification, Labelling and Packaging (CLP) system. This set of compounds comprised only 1 substance in category 1 (LD_50_ < 5 mg/kg b.w.), 7 substances in category 2 (5 < LD_50_ < 50 mg/kg b.w.), 12 substances in category 3 (50 < LD_50_ < 300 mg/kg b.w.), 20 substances in category 4 (300 < LD_50_ < 2000 mg/kg b.w.) and 11 substances in the non classified (NC) (LD_50_ > 2000 mg/kg b.w.) category. Two pharmaceutical drugs (maprotiline and chlormethiazole) could not be classified, as they had no rodent LD_50_ available.

### 2.2 Rodent LD_50_, human LC_50_ and LUCS EC_50_ data collection

The human LC_50_s (the 50 % Lethal Concentrations) were taken from a study by Sjöström et al. [40] who calculated them from sub-lethal and lethal blood concentrations derived from nearly 2800 human poisoning cases and compiled within the ACuteTox project (www.acutetox.eu). The LUCS EC_50_ data were collected from a previous study on human hepatic cells (HepG2) [41]. Mean *in vivo* oral rodent LD_50_s were extracted from two EURL ECVAM’s published studies: 1) Hoffmann et al. [26], a statistical review of compiled rat and/or mouse data, providing means of LD_50_ values (estimated from 10 and 20 individual values for most compounds) and where the principal sources of the *in vivo* animal data from oral acute toxicity studies were the internet databases ChemIDplus (http://chem.sis.nlm.nih.gov/chemidplus) and HSDB Hazardous Substances Data Bank (http://toxnet.nlm.nih.gov/newtoxnet/hsdb.htm), and 2) Kinsner-Ovaskainen et al. [42], providing rat LD_50_ data only. Finally, the LD_50_ values available for the 53 compounds selected in our study were presented as three datasets, named “Mean of rat oral LD_50_ from Hoffman et al., 2010″, “Mean of rat oral LD_50_ from Kinsner et al., 2013″, and “Mean of mouse oral LD_50_ from Hoffman et al., 2010)” (Table 1).

**Table 1:** Collection of acute toxicity data used for linear regression analyses.

Chemical	ACuteTox Nb	EU LCP acute oral toxicity category	Mean of Rat Oral LD_50_ Hoffmann et al, 2010		Mean of Rat Oral LD_50_ Kinsner-Ovaskainen et al, 2013		Mean of Mouse Oral LD_50_ Hoffmann et al, 2010		Human LC_50_ (Log M) Sjöström et al, 2008	LUCS EC_50_ Derick et al, 2017		3T3 NRU IC_50_ (Log M) Sjöström et al, 2008
			(mg/kg)	(Log M)	(mg/kg)	(Log M)	(mg/kg)	(Log M)		(Log M)	(SD)	
Acetaminophen	1	ND	2682.6	−1.75	2588.20	−1.77	667.60	−2.36	−2.66	−1.84	0.047	−3.48
Acetylsalicylic acid	2	4	1586	−2.06	1538.20	−2.07	1534.30	−2.07	−2.2	−2.14	0.058	−2.36
Caffeine	4	3	277.6	−2.84	278.60	−2.84	228.20	−2.93	−3.29	−1.93	0.057	−3.08
Carbamazepine	5	ND	3233.3	−1.86	2958.00	−1.90	2083.00	−2.05	−3.79	−3.11	0.101	−3.34
Isopropyl alcohol	10	ND	5075.4	−1.07	4954.50	−1.08	ND	ND	−0.94	−1.64	0.031	−1.2
Malathion	11	4	1276.1	−2.41	926.50	−2.55	1197.20	−2.44	−5.73	−3.41	0.019	−2.88
Mercury II chloride	12	2	53	−3.71	40.40	−3.83	29.80	−3.96	−4.71	−4.75	0.044	−4.8
Sodium valproate	16	4	858.3	−2.29	300.60	−2.74	848.10	−2.29	−2.2	−1.76	0.033	−2
5-Fluorouracil	17	3	ND	ND	230.10	−2.75	ND	ND	−3.69	−4.68	0.654	−4.3
Cadmium II chloride	21	3	152.6	−3.08	152.10	−3.08	ND	ND	−6.06	−5.82	0.024	−5.65
Amiodarone hydrochloride	28	ND	ND	−2.36	2999.20	−2.36	ND	ND	−4.95	−4.78	0.017	−4.58
Verapamil	29	3	ND	ND	100.00	−3.69	ND	ND	−5.21	−4.27	0.274	−4.14
Rifampicine	30	4	1797.5	−2.66	1644.40	−2.70	ND	ND	−3.81	−4.2	0.022	−3.99
Orphenadrine	32	4	ND	ND	328.90	−2.97	ND	ND	−4.64	−3.95	ND	−3.95
Lindane	34	3	157.6	−3.27	142.60	−3.31	210.00	−3.14	−5.98	−4.13	ND	−3.27
Ethanol	37	ND	12519.4	−0.57	11912.40	−0.59	8709.00	−0.72	−0.8	−1.61	0.027	−0.83
Parathion	38	2	8.4	−4.54	6.50	−4.65	14.10	−4.32	−5.65	−4.02	0.035	−3.67
Dichlorvos	39	3	64.3	−3.54	58.10	−3.58	95.30	−3.37	−3.7	−3.48	0.059	−3.77
Glufosinate amonium	41	4	1682	−2.07	1686.60	−2.07	436.70	−2.66	−1.99	−2.82	0.403	−2.12
Cis -diaminedichloroplatinum	42	2	ND	ND	25.80	−4.07	ND	ND	−4.68	−4.03	0.091	−5.25
Diquat dibromide	44	3	264.9	−3.11	209.90	−3.21	ND	ND	−3.55	−4.58	0.096	−4.38
Cyclosporine A	46	4	ND	ND	1485.90	−2.91	ND	ND	−6.22	−5.13	ND	−4.38
Sodium fluoride	48	3	161.2	−2.41	140.30	−2.48	106.90	−2.59	−3.24	−2.26	0.106	−2.72
Paraquat	49	3	124.6	−3.31	ND	ND	ND	ND	−5.02	−4.15	0.011	−4.07
Dimethyl formamide	51	ND	4151.5	−1.25	4111.50	−1.25	4579.90	−1.20	−2.23	−2.08	0.017	−1.14
Amitriptyline	53	4	406.7	−2.89	389.90	−2.91	219.90	−3.15	−5.34	−4.46	0.250	−4.64
Ethylene glycol	54	ND	8357.6	−0.87	7816.30	−0.90	9901.80	−0.80	−1.5	−1.8	0.092	−0.38
Phenol	56	4	572.7	−2.22	ND	ND	284.00	−2.52	−3.41	−3.13	0.017	−3.12
Sodium chloride	57	ND	4175	−1.15	4217.00	−1.14	5120.00	−1.06	−1.27	−1.11	0.035	−1.14
Potassium cyanide	59	2	9.22	−3.85	ND	ND	11.60	−3.75	−3.89	−2.85	0.065	−3
Lithium sulfate	60	4	ND	ND	1191.40	−1.96	ND	ND	−2.25	−1.25	ND	−1.81
Theophylline	61	3	ND	ND	ND	ND	276.00	−2.81	−3.29	−1.82	0.031	−3.06
Propanolol	63	4	ND	ND	625.20	−2.67	390.20	−2.88	−4.95	−4.22	0.067	−4.27
Arsenic trioxide	64	3	91.6	−3.33	49.70	−3.60	105.60	−3.27	−5.23	−4.69	0.025	−4.87
Thioridazine	65	4	1085	ND	ND	ND	409, 1	ND	−4.76	−4.98	0.074	−4.19
Thallium sulfate	66	2	21.5	−4.37	21.00	−4.38	ND	ND	−5.09	−4.09	0.067	−4.8
Warfarin	67	2	17	−4.26	6.30	−4.69	634.20	−2.69	−3.81	−3.02	0.035	−3.09
Isoniazid	69	ND	ND	ND	ND	ND	ND	ND	−3.35	−2.44	0.189	−1.83
Chloroquine	72	ND	ND	ND	ND	ND	ND	ND	−4.88	−4.4	0.058	−4.64
Chloramphenicol	74	ND	ND	ND	ND	ND	2270.00	−2.15	−3.35	−2.53	0.071	−3.14
Potassium chloride	75	ND	2015	−1.57	ND	ND	ND	ND	−1.98	−1.09	0.022	−1.05
Chloral hydrate	76	4	720.3	−2.36	638.30	ND	ND	ND	−3.18	−2.33	0.049	−2.95
2,4-Dichlorophenoxyacetic acid	77	4	378.9	−2.77	294.40	−2.88	366.50	−2.78	−2.43	−2.56	0.016	−2.87
Strychnine	80	2	13	−4.41	9.00	−4.57	ND	ND	−5.12	−2.82	0.027	−3.19
Maprotiline	82	ND	ND	ND	ND	ND	ND	ND	−5.53	−4.64	0.502	−4.7
Disopyramide	83	4	ND	ND	ND	ND	387.00	−2.94	−4.06	−2.78	0.043	−2.57
Chlormethiazole	85	ND	ND	ND	ND	ND	ND	ND	−3.49	−2.49	0.022	−2.85
Quinidine	86	4	ND	ND	ND	ND	584.70	−2.74	−4.52	−4.09	0.016	−4.26
Procainamide	87	4	ND	ND	ND	ND	811.70	−2.46	−3.24	−2.63	0.123	−2.79
Chlorpromazine	89	4	322.4	−3.04	ND	ND	342.40	−3.02	−6.46	−4.95	0.326	−4.82
Sodium selenate	91	1	ND	ND	3.10	−4.78	ND	ND	−4.59	−2.99	ND	−3.75
Acetonitrile	92	ND	3940	−1.02	3630.80	−1.05	ND	ND	−2.82	−0.66	0.025	−0.68
Sodium bicarbonate	93	ND	5555	−1.18	ND	ND	ND	ND	−0.96	−2.04	ND	−1.03

### 2.3 Materials and reagents

Thiazole orange (TO), H_2_0_2_, and the 20 chemicals used for the LUCS study were purchased from Sigma-Aldrich (Saint-Quentin Fallavier, France). Gibco DMEM (high glucose, GlutaMAX supplement and pyruvate), fetal bovin serum (FBS) (HyClone), pen-strep solution (100X) (Gibco), 0.05 % Trypsin-EDTA (HyClone), Gibco DPBS without Calcium and Magnesium (1X), and were purchased from Thermo Fisher Scientific. HepG2 cell line was purchased from the American Type Cell Collection (ATCC) (LGC Standards, Molsheim, France), catalog number HB8065.

### 2.4 Cell culture

HepG2 cells (passages 15–35) were cultured at 37 °C/5% CO_2_ in DMEM medium complemented with 10 % FBS and 1% pen-strep solution. Cells were grown up to 70–80 % confluence then transferred in clear bottom 96-well microplates at a density of 10^6^ cells/ml (75 μL, 75,000 cells/well) and cultured in the same culture medium for 24 h.

### 2.5 Dose-response experiments

Compound treatment or cell dye labelling were performed in serum-free medium to avoid interaction with serum components. Cells were incubated for 24 h with each of the 20 tested compounds under eight different concentrations obtained by factor 2 or 3.16 serial dilutions. Solvent was always ≤ 1% (vol/vol) in the highest compound concentration assayed and maintained at the same concentration during the dilution process.

### 2.6 LUCS (Light-Up Cell System) assays

#### 2.6.1 “Light-Induced” LUCS version (L-LUCS)

The mechanistic model of LUCS has been described elsewhere [41]. Briefly, when fluorescent cyanine dyes such as Thiazole Orange (TO) or SYTOs are added in the cell culture medium, they are mainly removed out of the cell by efflux transport, limiting their access to nucleic acid targets, resulting in low fluorescence. Application of light induces intracellular ROS production that alters efflux and/or other cell functions leading to massive entry of TO or SYTO dyes, triggering increase in fluorescence emission.

At the end of the 24 h compound treatment, HepG2 cells were incubated for 30 min at room temperature with 4 μM TO. Fluorescence intensity (*i.e.* F_pre_) was measured using a Varioskan Flash Spectral Scanning Multimode Reader (Thermo Fisher Scientific, Waltham, Mass., USA) set up at 505/535 nm (excitation/emission wavelengths). The 96-well microplates were then placed in a dedicated illuminator device based on 24 LEDs (470 nm), each one centered on the intersection of 4 wells, and illuminated at 24 mW/cm^2^ during 10 s. The fluorescence was measured a second time (*i.e.* F_post_) immediately after illumination.

#### 2.6.2 “Chemically-Induced” LUCS version (C-LUCS)

At the end of the 24 h compound treatment, HepG2 cells were incubated for 30 min at room temperature with 4 μM TO. The fluorescence intensity (*i.e.* F_pre_) was measured using a Varioskan Flash Spectral Scanning Multimode Reader (Thermo Fisher Scientific, Waltham, Mass., USA) set up at 505/535 nm (excitation/emission wavelengths). Cells were then incubated with 0.1 % H_2_O_2_ for 90 min. The fluorescence was measured a second time (*i.e.* F_post_).

### 2.7 EC_50_ and coefficient of determination (R^2^) evaluations

For dose-response experiments, F_post_/F_pre_ ratios obtained for each dose were fitted using GraphPad Prism software with a mathematical non linear regression model: Y = Bottom + (Top-Bottom)/(1 + 10^((LogEC_50_-X)*HillSlope)), where X is the measured value and HillSlope the slope of the straight line defining the tangent at the inflection point. 50 % efficacy concentration (EC_50_) and R^2^ values were calculated from the regression model.

## 3 Results & discussion

### 3.1 Data collection

Table 1.S. gives a description of the 53 chemicals selected from the ACuteTox databank. Data in this table were derived from the ACuteTox database and from [39], with compound use applications (pharmaceutical drugs, biocides, industrial chemicals), acute oral toxicity categories (assigned to the LD_50_ values according to the EU CLP regulation), production or absence of metabolites in humans, basal cytotoxicity and/or organ specific toxicity, and possible mechanisms involved in human acute toxic effects. As previously shown in [39], it appears that cytotoxicity is an important determinant of acute systemic toxicity, and that there is no clear relationship between specific mechanisms of target organ toxicity and the four categories of acute toxicity.

Table 1 gives a summary of all the collected data used for linear regression analyses: rat and mouse mean oral LD_50_ values from three datasets (“Mean of rat oral LD_50_ from Hoffman et al”, “Mean of rat oral LD_50_ from Kinsner et al” and “Mean of mouse oral LD_50_ from Hoffman et al”, [26,42]), human LC_50_ and mouse 3T3 (fibroblasts) NRU EC_50_ values collected from Sjöström et al. [40], and EC_50_ values obtained in human HepG2 cells (liver) using LUCS assay from Derick et al. [41].

### 3.2 Evaluation of human predictivity value of LUCS assay data

**Fig. 1:**
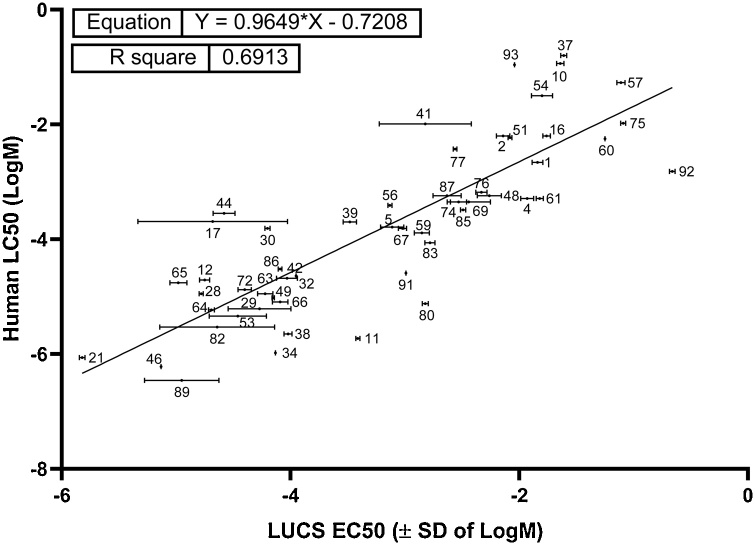
Comparison of cell-based LUCS and human acute poisoning data. Regression analysis of Human LC_50_s and LUCS EC_50_s obtained from dose-response experiments using 53 out of the 97 substances of the ACuteTox European Program databank, showing that LUCS predicts human acute oral toxicity to 69 %. The corresponding SD error bars are indicated for log-transformed LUCS EC_50_ data.

The relevance of cell-based test data for acute human toxicity has been inferred previously by the MEIC study. LUCS acute toxicity EC_50_ (logM) data generated from our previous study [41] and human acute LC_50_ (logM) data from the ACuteTox programme [40] were compared. Analysis of correlation by linear regression was possible for the 53 chemicals common in the two databases, and resulted in a determination coefficient (R^2^) value of 0.691 with a regression line slope = 0.965, close to normal value (Fig. 1). This result indicated that LUCS assays predicted human oral acute toxicity to 69 %.

In addition, according to compound classification (pharmaceutical drugs, industrials, and biocides/pesticides, Table S1), the chemicals with higher human to *in vitro* differences seem to belong to specific categories. The 20 % (11/53) of chemicals that were the more over- or under-predicted with LUCS test only belong to two categories, biocide/pesticide (7/11, malathion, lindane, parathion, strychnine, sodium selenite, glufosinate ammonium, diquat dibromide) (63.6 %) and industrial (4/11, acetonitrile, isopropylalcool, ethanol, sodium bicarbonate) (36.4 %) groups. None of them belong to the drug group (0/11) although this latter is the most represented (30/53) over the three groups. The over-representation of biocides/pesticides in compounds that show the most human to *in vitro* differences might be due to specific properties such as low water solubility, low cellular metabolization/detoxification level and/or species-specific functions.

### 3.3 Comparison of LUCS *versus* rodent data to predict human acute toxicity

**Fig. 2:**
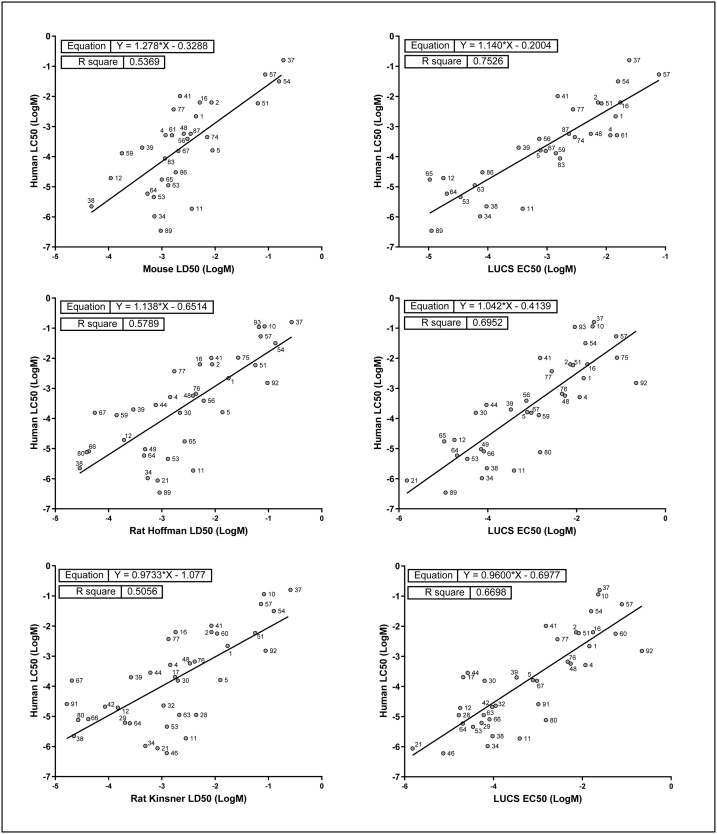
Comparative evaluation of animal *versus* LUCS data to predict human acute oral toxicity. Regression analysis of human oral toxicity (LC_50_, vertical axis) and animal-based (LD_50_, horizontal axis, left side) or LUCS cell-based (EC_50_, horizontal axis, right side) data performed using data sets restricted to compounds for which common data were available.

In order to evaluate which tests predict human toxicity the best, we then compared the different R^2^ values obtained for regressions of LUCS with human LC_50_ *versus* rodent data with human LC_50_, calculated for each of the three rodent LD_50_ datasets (Table 2). Only chemicals with shared data were retained for analyses. All linear regression analyses are depicted in Fig. 2. Using a first rat dataset (from [42]) we identified 37 substances with both LUCS and rat LD_50_ data, and found for linear regression analysis of LUCS with human acute toxicity an R^2^ = 0.670 (regression line slope = 0.96), and for regression of rat with human acute toxicity an R^2^ = 0.504 (regression line slope = 0.97). Using a second rat dataset (from [26]), we identified 35 substances with both LUCS and rat LD_50_ data, and found for regression of LUCS with human acute toxicity an R^2^ = 0.695 (regression line slope = 1.04) and for regression of rat with human acute toxicity an R^2^ = 0.579 (regression line slope = 1.14). Eventually, from the mouse dataset [26], we identified 30 substances with both LUCS and mouse data, and found for regression of LUCS with human acute toxicity an R^2^ = 0.753 (regression line slope = 1.14) and for regression of mouse with human acute toxicity an R^2^ = 0.537 (regression line slope = 1.28). The R^2^s for regression of LUCS with human data were always higher than the R^2^s for regression of rodent with human data, suggesting that the LUCS test predicts human acute toxicity better than standard *in vivo* animal tests do: 67 % *versus* 50.4 % or 69.5 % *versus* 57.9 % for rat data, and 75.3 % *versus* 53.7 % for mouse data. LUCS-based regression line slopes were closer to normal value than corresponding animal-based ones.

**Table 2:** Comparative analysis of LUCS and animal-based acute toxicity tests to predict human acute toxicity. In all cases, compounds used for linear regression analysis (n) correspond to compounds for which data exist for both tests. ^a^ [41]; ^b^ [42]; ^c^ [26]; in bold: best of the two values (R^2^ closer to 1, slope closer to 1, intercept closer to 0).

Comparison	n	Test	R^2^ (test *vs* human)	Slope	Intercept
LUCS^a^ & rat^b^ data	37	LUCS (HepG2 cells)	**0.670**	0.960	**−0.698**
		Rat	0.504	**0.971**	−1.089
LUCS^a^ & rat^c^ data	35	LUCS (HepG2 cells)	**0.695**	**1.042**	**−0.414**
		Rat	0.579	1.138	−0.651
LUCS^a^ & mouse^c^ data	30	LUCS (HepG2 cells)	**0.753**	**1.140**	**−0.200**
		Mouse	0.537	1.279	−0.324

Furthermore, the R^2^ value obtained for human *versus* LUCS data (R^2^ = 0.691, regression line slope = 0.96, n = 53) was similar to the one for human *versus* 3T3 NRU data taken from [40] (R^2^ = 0.694, regression line slope = 0.93, n = 53).

These results suggest that the LUCS test is more effective than rodent acute oral toxicity test methods in predicting human acute oral toxicity. This is in line with the MEIC programme which studied the relevance of cell-based assays and rodent oral acute toxicity LD_50_ for predicting acute human toxicity [[30], [31], [32],^43^]. MEIC study showed that rat LD_50_ values predicted human data (LC, human acute lethal oral concentrations) to 60.7 % (R^2^ = 0.607, slope 0.83, n = 50) and that mouse LD_50_ values predicted human data to 65.3 % (R^2^ = 0.653, slope 0.90, n = 50). The same set of 50 substances were tested in 61 different *in vitro* assays (among which, 20 human cell assays, 21 non-human mammalian cell assays, 18 ecotoxicological tests, and 2 cell-free systems). Partial least squares (PLS) analysis indicated that the data generated from the 61 tests predicted human data to 80 % (R^2^ = 0.80) and that human cell line assays predicted human data to 74 % (R^2^ = 0.74).

### 3.4 Optimization of LUCS test method: light- *versus* chemically-induced LUCS protocoles

**Fig. 3:**
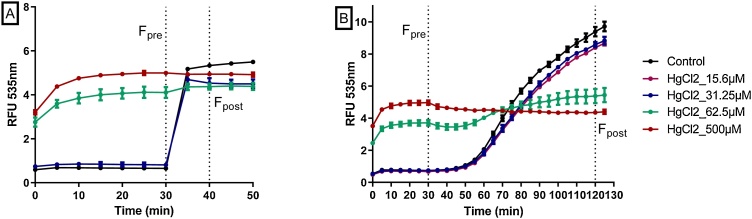
Optimization of LUCS experimental procedure. A new experimental procedure based on H_2_O_2_ treatment (B) was developed to avoid the sample illumination step (A) classically used in LUCS assay. Cells were treated with increasing doses of mercury chloride for 24 h, then with TO at 4μM final concentration for 30 min ; (A) plates were exposed to a LED based light source (0.24 J/cm^2^ centered at 470 nm); (B) plates were treated with 0,1% H_2_O_2_. Fluorescence was measured at 535 nm. F_pre_ and F_post_ represent the fluorescence values retained for homeostasis index determination.

The LUCS test method as depicted in Derick et al. (2017) [41] uses a homemade LED device as light source for photo-induction. An example of fluorescence kinetics record obtained for both light- and chemically-induced LUCS protocoles after 24 h treatment with mercury (II) chloride is given in Fig. 3: in both cases, TO F_pre_ values are high under toxic conditions and low under non-toxic ones, while F_post_ values remained almost unchanged whatever mercury (II) chloride concentration is applied, showing that F_post_/ F_pre_ ratios can be used as an indicator of cell toxicity.

**Fig. 4:**
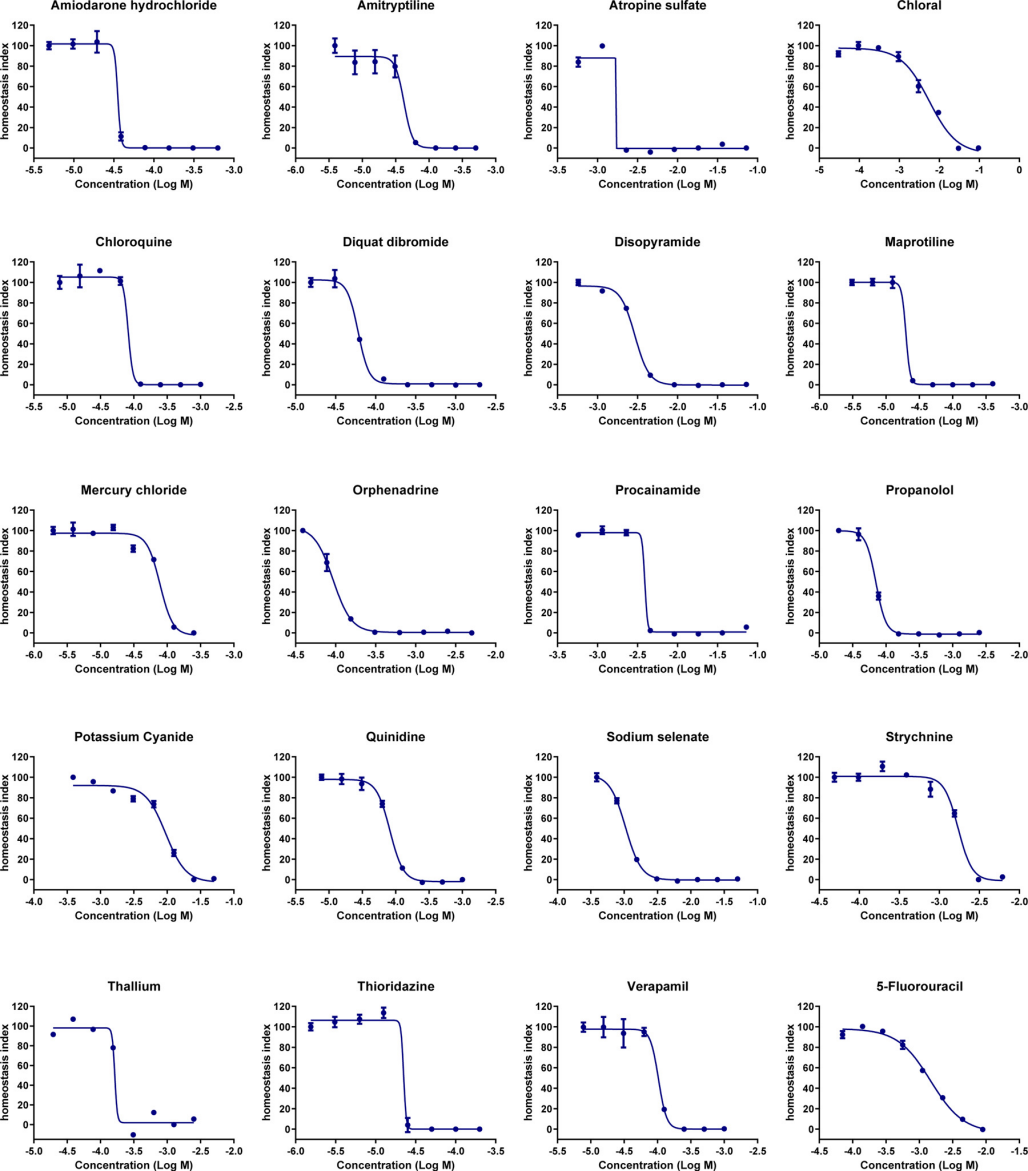
Homeostasis dose-response curves of 20 selected compounds determined by light-induced LUCS. Homeostasis indices (HI) were calculated from F_pre_ and F_post_ values as shown in Fig. 3A in the case of mercury chloride, using the formula HI_x_ = (Q_x_-Q_min_)/Q_max_-Q_min_) with Q_x_ = (F_x post_/F_x pre_)x100. Each of the EC_50_s are reported in Table 3.

**Fig. 5:**
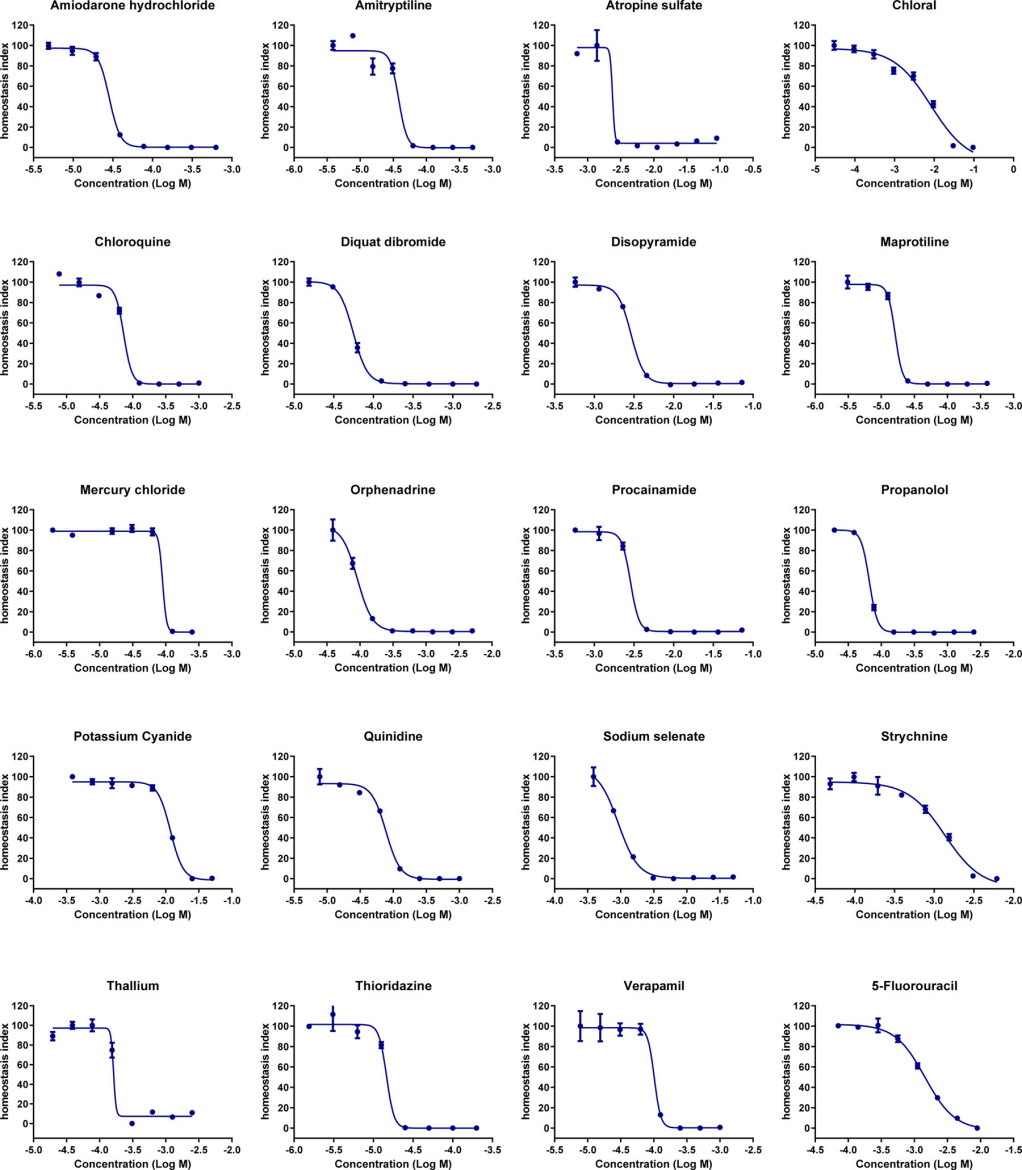
Homeostasis dose-response curves of 20 selected compounds determined by H_2_0_2_-induced LUCS. Homeostasis indices (HI) were calculated from F_pre_ and F_post_ values as shown in Fig. 3B in the case of mercury chloride, using the formula HI_x_ = (Q_x_-Q_min_)/(Q_max_-Q_min_) with Q_x_ = (F_x post_/F_x pre_)x100. Each of the EC_50_s are reported in Table 3.

**Fig. 6:**
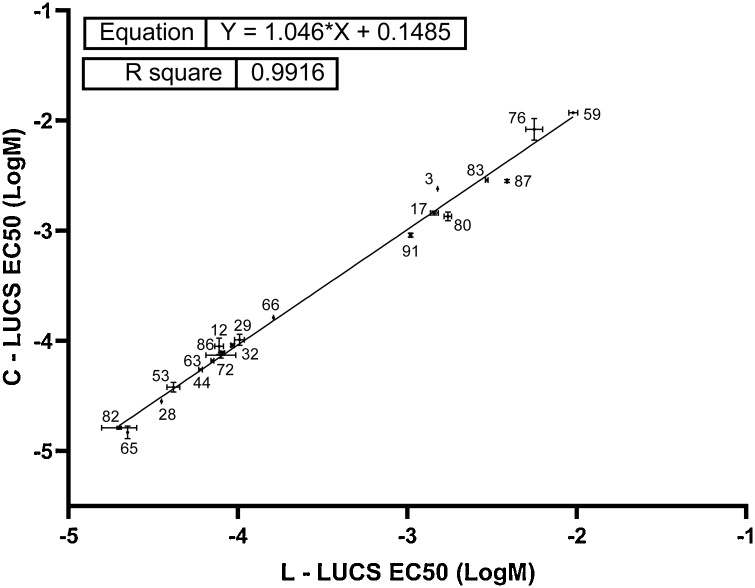
Comparison of C-LUCS *versus* L-LUCS EC_50_s. Regression analysis of light-based (L-LUCS) and chemically H_2_O_2_-based (C-LUCS) EC_50_s obtained from dose-response experiments using a selection of 20 substances of the ACuteTox European Program databank, showing that the two procedures predict each other to 99 %.

LED device used for light-induced LUCS (L-LUCS) is not commercially available which may limit implementation of the method by end users. An alternative method was developed by replacing the photoinduced ROS production by addition of H_2_O_2_ in the culture medium. This new protocol was called C-LUCS for chemically-induced LUCS. L-LUCS and C-LUCS procedures were tested on HepG2 cells using a set of 20 compounds from AcuteTox database. Fig. 4, Fig. 5 depict dose-response curves obtained with L-LUCS and C-LUCS protocols, respectively. The list of the 20 compounds and the corresponding EC_50_ values obtained using the different protocols are given in Table 3. Linear regression analysis of the results obtained from the two protocols gave a slope = 1.046, close to normal, and an R^2^ = 0.992 (Fig. 6) demonstrating the ability of the C-LUCS protocol to be used in the L-LUCS test’s homeostasis/toxicity application fields. This result also makes C-LUCS protocol an easy option as a fast tool for acute toxicity screening in any existing HTS platform.

**Table 3:** EC_50_s, log EC_50_s and R^2^s obtained from analyses of the sigmoid fits shown in fig. 4 & 5, according to the two different LUCS experimental procedures. *R^2^* ≥ 0.97 was retained as quality control criterium. Experiment were redone until R^2^s obtained for both L- and C-LUCS reached this value. ND = not determined following ambiguous sigmoid fit.

Chemical (A–Z)	L-LUCS				C-LUCS			
	EC_50_ (M)	EC_50_ (Log M)	R^2^	SD	EC_50_ (M)	EC_50_ (Log M)	R^2^	SD
Amiodarone hydrochloride	3.59E-05	−4.45	0.99	ND	2.85E-05	−4.55	0.99	0.009481
Amitriptyline	4.21E-05	−4.38	0.97	0.03777	3.82E-05	−4.42	0.97	0.04454
Atropine sulfate	1.53E-03	−2.82	0.99	ND	2.38E-03	−2.62	0.98	ND
Chloral	5.60E-03	−2.25	0.98	0.04975	8.30E-03	−2.08	0.97	0.0987
Chloroquine	8.22E-05	−4.11	0.99	0.08846	7.34E-05	−4.13	0.98	0.02629
Diquat dibromide	6.04E-05	−4.22	0.99	0.0106	5.56E-05	−4.26	0.99	0.006953
Disopyramide	2.94E-03	−2.53	0.99	0.007675	2.91E-03	−2.54	0.99	0.008145
Maprotiline	2.00E-05	−4.7	0.99	0.1037	1.62E-05	−4.79	0.99	0.01437
Mercury chloride	7.80E-05	−4.11	0.98	0.02529	8.93E-05	−4.05	0.99	0.0752
Orphenadrine	9.27E-05	−4.03	0.99	0.009865	9.13E-05	−4.04	0.99	0.01367
Procainamide	3.91E-03	−2.41	0.99	ND	2.85E-03	−2.55	0.99	0.01494
Propanolol	7.06E-05	−4.15	0.99	0.008178	6.61E-05	−4.18	0.99	0.006696
Potassium Cyanide	9.51E-03	−2.02	0.98	0.02568	1.17E-02	−1.93	0.99	0.009136
Quinidine	8.22E-05	−4.09	0.99	0.01061	7.75E-05	−4.1	0.99	0.01543
Sodium Selenate	1.05E-03	−2.98	0.99	0.006627	9.20E-04	−3.04	0.99	0.0179
Strychnine	1.72E-03	−2.76	0.97	0.02236	1.36E-03	−2.87	0.98	0.03863
Thallium	1.64E-04	−3.79	0.98	ND	1.64E-04	−3.79	0.98	ND
Thioridazine	2.23E-05	−4.65	0.99	ND	1.50E-05	−4.83	0.98	0.05861
Verapamil	1.03E-04	−3.99	0.99	0.02866	1.01E-04	−3.99	0.98	0.04953
5-Fluorouracil	1.46E-03	−2.84	0.99	0.02371	1.45E-03	−2.84	0.99	0.01857

Some differences appear between EC_50_s calculated from [41] and the present study. They are due to an optimization of LUCS protocol. SYTO dye was used as photo-inducer in the former study. It was replaced by TO in the present study because the latter gives a better signal amplitude in terms of fluorescence variations when comparing normal and toxic conditions. Linear regression analysis of SYTO *vs* TO LUCS data gives an R^2^ of 0.766, increasing to 0.929 when 5-fluorouracil (compound 17), which showed a very atypical dose-response profile and high SD value in the SYTO study, is removed from the analysis.

## 4 Conclusions

This study shows that data generated with the *in vitro* cell-based LUCS test resulted in a better prediction of human acute toxicity data than the *in vivo* rodent oral acute toxicity tests do. Meigs et al. [44] have already shown that animal tests are costly, time-consuming, unethical, and give misleading results. They have discussed the important positive aspects of alternative methods for industry (more effective and rapid discovery of new entities, increased satisfaction of the end users with regards to the 3Rs, manufacturing of better and safer products). By creating a large toxicological database from ECHA’s data (aproximately 10,000 chemicals), Luechtefeld et al. [45] could assess the reproducibility of the animal tests used and showed that *in vivo* OECD tests were not strongly reproducible [46]. The animal OECD tests are furthermore not always relevant. In the case of the AOP-based *in vitro* skin sensitization testing, analysis of results from *in vivo* mouse local lymph node assay (LLNA) – which are used as reference test for evaluating predictive capacity of new alternative methods – revealed a great variability leading to a discordant hazard classification [47,48]. In a study by Urbisch et al. [49], the non-animal test methods predicted human data more accurately (90 %) than LLNA data (82 %). Also, in the case of serious eye damage/irritation testing, uncertainties associated with animal-to-animal variability within-test were reported for the Draize eye test performed in rabbit, leading to *in vivo* misclassification [50].

The concept of the adverse outcome pathway (AOP) used to represent the mechanistic events leading to an adverse outcome in an animal or population is based on the identification of molecular initiating events (MIEs) or key events (KEs). For acute systemic toxicity, it was suggested by Vinken and Blaauboer [51] to consider *in vitro* basal cytotoxicity as a first step of a tiered strategy in evaluating the toxicity of chemicals. The authors proposed a generic AOP named « from chemical insult to cell death » consisting of three steps (initial cell injury, mitochondrial dysfunction, and cell demise). Toxicity may be related to multiple critical pathways indeed, reflected by the description of a continuously increasing number of AOPs.

In contrast, the number of cellular stress response pathways through which cells respond to exogeneous stresses (toxic chemicals, xenobiotics, heat, radiation) by transcriptional activation of cytoprotective genes and which also participate in cell fate decisions, is quite limited [52]. Under toxic compound exposure, the perturbations of functional pathways may trigger the cells under stress to establish a new homeostasis (cell adaptation); alternatively, efforts aimed at restoring homeostasis could fail and lead to cell death (or adversity). Hence, the LUCS test method, which allows evaluation of whether the cells still have the capacity for a recovery after a potential loss of homeostasis caused by chemicals after 24 h treatment [41], is a good candidate for assessing their status in a regulatory context. Whatever LUCS protocol is applied, the test takes advantage of fluorescent nucleic acid-binding asymmetrical cyanines as intracellular sensors. Intracellular events explaining pre- and post- fluorescence levels have been previously investigated and partially unveiled. Cyanine sensors have been shown to be substrates of proton/cation antiporters of the MATE or MSF plasma membrane transport protein families [41]. For cells in homeostasis, antiporters remove cyanin biosensors from the cell with only a small part of them being able to bind their nucleic acid target leading to low fluorescence intensity. When cells are damaged after a treatment with toxic compounds (alternatively after light application in the case of l-LUCS or H_2_O_2_ treatment in the case of C-LUCS), dysfunction of antiporters triggers massive passage of the sensor through the plasma membrane, saturating nucleic acid binding sites, leading to high fluorescence intensity.

Overall, LUCS test method measures accurate signals of cell homeostasis on a high throughput format with a live cell readout that keeps cells operational for parallel or further analyses (multiplexing). In addition, the LUCS test presents the benefit of adaptability to any cell lines (assuming they contain DNA), including iPSCs. Ratio mode of analysis also allows quantitative data that remain to a certain extent independent of the number of cells present in the well.

This work also aimed to show that the LUCS can be optimized towards a universal protocol adapted to end users equipped with fluorescence readers or high throughput platforms. Robustness of the LUCS test has been evaluated earlier (Derick et al., 2017). Inter-microplate Z’ value of 0.8 (calculated for 1344 wells) showed than LUCS is suitable for high throughput applications. The LUCS test method has been previously used successfully by other laboratories working on nanomaterials [53] and marine toxins [54]. L-LUCS test can be performed using commercial fluorescence readers as a source of energy but it would take 15 min to get the result for each experimental condition. The time can be strongly reduced (<10 s) by using an appropriate LED illumination device. Because a dedicated device might limit implementation of the method, a second version of the test method was developed with the addition of a chemical agent involved in ROS production, thus mimicking photo-induction process. LUCS readout, characterized by a huge increase of cell fluorescence in the case of toxicity, is another important issue for multiplex analysis as it can be easily differentiated from other signals such as substance intrinsic fluorescence for instance, that tend to drop upon illumination. This clearly adds value to the LUCS test.

Thus, the integratation of LUCS test as a new tool in toxicological regulatory procedures and HTS platforms would allow a new quantitative read-out prioritising chemicals for more detailed studies or replacement of current *in vivo* tests within a tiered strategy. For example, by building *in vitro* testing batteries allowing toxicology testing for three key target organs (*i.e.* heart, liver, CNS). The present level of standardization of the LUCS test method allows us to envisage its implementation onto organs-on-a-chip, 3D cultures in toxicology testing (proof of concept on bio-printed human skin models was recently demonstrated, unpublished results). One of the most promising next step is to use the universality of LUCS in term of cell models by applying the technology to differentiated cells from induced pluripotent stem cells (iPSCs) in order to target complex regulatory applications such as oral, dermal and inhalation acute toxicities, which still necessitate animal tests.

## Author contributions

C.F. designed the LUCS test and revised the manuscript. C.G. carried out LUCS experiments. C.F. and C.D. performed regression and other analyses. C.D. compiled data and wrote the manuscript.

## Declaration of Competing Interest

The authors declare the following financial interests/personal relationships which may be considered as potential competing interests:

CF is co-inventor in a patent on LUCS technology. CG, CD & CF are also employees of Anti Oxidant Power (AOP).
